# Self-soothing behaviors with particular reference to oxytocin release induced by non-noxious sensory stimulation

**DOI:** 10.3389/fpsyg.2014.01529

**Published:** 2015-01-12

**Authors:** Kerstin Uvnäs-Moberg, Linda Handlin, Maria Petersson

**Affiliations:** ^1^Department of Animal Environment and Health, Swedish University of Agricultural Sciences, Skara, Sweden; ^2^School of Health and Education, University of Skövde, Skövde, Sweden; ^3^Department of Molecular Medicine and Surgery, Endocrine and Diabetes Unit, Karolinska Institutet, Stockholm, Sweden

**Keywords:** oxytocin, non-noxious sensory stimulation, afferent nerves, anti-stress effects

## Abstract

Oxytocin, a hypothalamic nonapeptide, is linked to increased levels of social interaction, well-being and anti-stress effects. The effects of oxytocin that is released by sensory stimulation during different kinds of interactive behaviors are often underestimated or even forgotten. In fact, many of the positive effects caused during interaction, such a wellbeing, stress reduction and even health promotion, are indeed linked to oxytocin released in response to activation of various types of sensory nerves. Oxytocin is released in response to activation of sensory nerves during labor, breastfeeding and sexual activity. In addition oxytocin is released in response to low intensity stimulation of the skin, e.g., in response to touch, stroking, warm temperature, etc. Consequently oxytocin is not only released during interaction between mothers and infants, but also during positive interaction between adults or between humans and animals. Finally oxytocin is also released in response to suckling and food intake. Oxytocin released in the brain in response to sensory stimulation as a consequence of these types of interactive behaviors, contributes to every day wellbeing and ability to handle stress. Food intake or sex may be used or even abused to achieve oxytocin-linked wellbeing and stress relief to compensate for lack of good relationships or when the levels of anxiety are high. The present review article will summarize the role played by oxytocin released by sensory (in particular somatosensory) stimulation, during various kinds of interactive behaviors. Also the fact that the anti-stress effects of oxytocin are particularly strong when oxytocin is released in response to “low intensity” stimulation of the skin will be highlighted.

## INTRODUCTION

Human individuals express different behaviors in order to feel well and to avoid tension and stress. Some of these behaviors are maladaptive and could be regarded as expressions of abuse, whereas others clearly represent healthy and natural ways of achieving every day wellbeing and relief from stress. A common denominator of several of the natural “soothing mechanisms” is that they often involve some type of sensory stimulation of skin or mucosa. Oxytocin, released within the brain from oxytocinergic nerves emanating from the paraventricular nucleus (PVN) in response to such sensory stimuli, is of crucial importance for the positive effects linked to these self-soothing behaviors (Buijs, 1983; Buijs et al., 1983; Sofroniew, 1983; Uvnäs-Moberg, 1998).

Oxytocin may, e.g., induce wellbeing by stimulation of dopamine release in the nucleus accumbens (NA) (Insel, 2003), increase social interaction and decrease anxiety by actions in the amygdala (Amico et al., 2004), decrease stress reactions by actions in the hypothalamic-pituitary—adrenal axis (HPA-axis) (Petersson et al., 1999b; Neumann, 2002) and by decreasing noradrenergic release in the locus coeruleus (LC) (Petersson et al., 1998b) and nucleus tractus solitarius (NTS) (Petersson et al., 2005a). Oxytocin may also decrease the sensitivity to pain by increasing opioidergic activity in the periaqueductal gray (PAG) (Lund et al., 2002). Oxytocin also modulates serotoninergic activity (Yoshida et al., 2009).

Oxytocin is released in response to activation of sensory nerves (Stock and Uvnäs-Moberg, 1988) not only during labor and breastfeeding, but also in response to skin-to-skin contact between mothers and infants (Matthiesen et al., 2001), during sexual intercourse (Carmichael et al., 1987) in both sexes, in connection with positive, warm interactions between humans (Light et al., 2005) and interaction between humans and animals (in particular dogs; Odendaal and Meintjes, 2003; Miller et al., 2009; Handlin et al., 2011), in response to several kinds of massage (Uvnäs-Moberg, 2004) and even in response to suckling (Lupoli et al., 2001) and food intake (Ohlsson et al., 2002).

The present article will be restricted to self-soothing mechanisms linked to oxytocin release in response to sensory stimulation and, in particular, to somatosensory stimulation. This is not to restrict the importance of stimuli mediated by the other senses or other types of mental activity, but since these mechanisms are often overlooked. The noxious and the non-noxious information mediated by peripheral sensory nerves play an important role by informing us about the state of the internal and external environment during our entire life. First some important aspects of the oxytocin system based on observations in animals and humans will be described, including the link between sensory stimulation and oxytocin release and oxytocin induced effects, in particular anti-stress effects. Thereafter the role of oxytocin release in basic female reproductive situations, such as birth (including skin-to-skin contact after birth) and breastfeeding, will be described, since some important information regarding the interactive effects between oxytocin release and sensory stimulation can be obtained from these models. The role of oxytocin and sensory stimulation for the positive consequences of other types of human interaction, e.g., sexual interaction, warm, and positive relationships including relationships between humans and dogs, will be described. Finally we will describe the effects of activation of sensory nerves and oxytocin release in connection to various types of massage or tactile stimulation, suckling and food intake.

All these “interactive behaviors” give rise to wellbeing and reduced stress levels. As oxytocin is involved, bonding to the source of the interaction may develop. In addition oxytocin release caused by the interactive procedures described above will stimulate mechanisms related to restoration and healing and therefore in a more long-term perspective they will contribute to a better health profile and longevity. For most people the above listed self-soothing behaviors are part of everyday normal life and contribute to wellbeing and relaxation. It is, however, becoming clear that some individuals “overuse” some of these self-soothing behaviors. There are many reasons for a need of such an “overdose,” but individuals who lack positive social relationships or have a low function in their oxytocin system, e.g., those with attachment disorders, may need to use one or several of the above listed self-soothing behaviors more than others in order to restore their oxytocin function and to feel well and relaxed. In some individuals the “overuse” may reach abusive levels.

## OXYTOCIN

### CHEMICAL, MORPHOLOGICAL, AND FUNCTIONAL ASPECTS

Oxytocin is a small peptide consisting of only nine amino acids and is produced in two nuclei within the hypothalamus, i.e., the PVN and the supraoptic nucleus (SON). From magnocellular neurons within the PVN and SON oxytocinergic neurons project to the neurohypophysis wherefrom oxytocin is released into the circulation acting as a classical hormone, mediating uterine contraction during labor and milk ejection during breastfeeding (Burbach et al., 2006).

**FIGURE 1:**
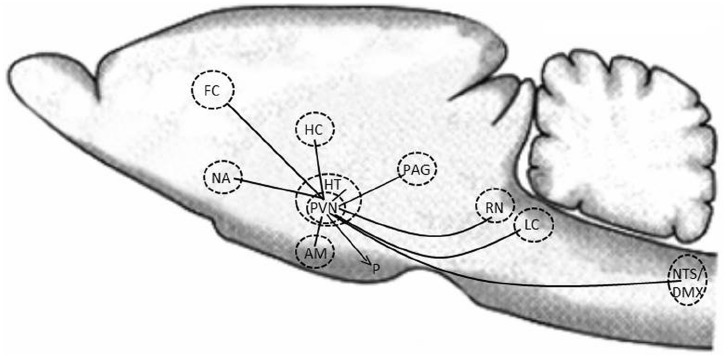
**Schematic illustration of how oxytocinergic neurons within the PVN project to some important regulatory areas in the CNS.** HC, hippocampus; HT, hypothalamus; PVN, paraventricular nucleus; NA, nucleus accumbens; AM, amygdala; PAG, periaqueductal gray; RN, raphe nuclei; LC, locus coeruleus; NTS, nucleus tractus solitarius; DMX, dorsal motor nucleus; FC, frontal cortex; P, pituitary.

However, oxytocin is also an important neurotransmitter within the brain. Parvocellular neurons from the PVN project to many important regulatory areas within the brain, e.g., other nuclei within the hypothalamus, the amygdala, the hippocampus, the PAG, the frontal cortex, the raphe nuclei, the striatum, the NA, the vagal nuclei (both the nucleus of the solitary tract (NTS) and the dorsal vagal nucleus (DMX), and the LC. Oxytocinergic neurons also reach the pineal gland, the cerebellum and the spinal cord (Figure 1) (Buijs, 1983; Buijs et al., 1983; Sofroniew, 1983; Knobloch et al., 2012; Stoop, 2012). Thus oxytocin reaches several important areas in the central nervous system (CNS), which are involved in the regulation of social interactive behaviors, fear, aggression perception of pain, calm, wellbeing, and stress reactions (by modulating the activity of the HPA-axis and the sympathetic and parasympathetic nervous system). Oxytocin can also be released from dendrites of the oxytocinergic neurons in the SON and PVN and then by diffusion and volume transmission reach distant locations in the brain to induce oxytocin mediated effects. For example such effects might occur when oxytocin is released in high amounts as, e.g., during parturition and breast-feeding (Ludwig and Leng, 2006; Fuxe et al., 2012). During these occasions high amounts of oxytocin is released in parallel both into the periphery and within the CNS (Keverne and Kendrick, 1994).

Oxytocin may act via one or several of these different mechanisms at the same time and also different combinations of oxytocin mediated effects might be activated. In this way separate oxytocin effects are integrated into broader effect patterns. Different patterns of oxytocin mediated effects are induced during labor, breastfeeding and social interactions involving touch (Uvnäs-Moberg and Petersson, 2010; Uvnäs-Moberg and Prime, 2013). As will be described in more detail below, when oxytocin release is induced by low intensity somatosensory stimulation, the anti-stress effect pattern becomes particularly prominent.

### OXYTOCIN RECEPTORS

The amount of oxytocin receptors and their binding properties are of course also of fundamental importance for the effects of oxytocin. Here both sex steroids and glucocorticoids play an important role since they both have the capacity to influence the expression of oxytocin receptors as well as the binding of oxytocin to receptors in the brain (Schumacher et al., 1993; Pfaff et al., 1999). Not all oxytocin-mediated effects are blocked by antagonists directed toward the uterine type of oxytocin receptor, e.g., some of the anti-stress, growth promoting and restorative effects of oxytocin. The reason is that oxytocin is metabolized and degraded into several smaller cyclic and linear oxytocin fragments (de Wied et al., 1987). A C-terminal fragment has been linked to calming, anti-stress, and growth promoting effects of oxytocin (Petersson et al., 1999c; Petersson and Uvnäs-Moberg, 2004). Variants of the oxytocin receptor gene have been demonstrated, some of which have been associated with different capabilities to recognize facial expression (Kumsta and Heinrichs, 2013). Some variants of the oxytocin receptor gene are also more prevalent in individuals with schizophrenia and autism (Montag et al., 2013; Yamasue, 2013).

### PHARMACOLOGICAL PROPERTIES

The half-life of oxytocin in the circulation of humans is 30 min (De Groot et al., 1995). A similar half-life has been demonstrated in the cerebrospinal fluid, but might be even longer in different parts of the brain (Jones and Robinson, 1982). The half-life of the oxytocin fragments is not known, but may be longer than that of the mother molecule. Only very small amounts of oxytocin in the circulation pass to the brain via the blood–brain barrier (BBB; <1%), but the permeability of the BBB may be increased during stress and in connection with certain types of disease. Oxytocin may also be transported across the BBB by specific carrier proteins (Jones and Robinson, 1982).

Oxytocin exerts some of its actions by modulating the function of other signaling systems. It, e.g., influences the release of dopamine in the NA (Insel, 2003), serotonin within serotonergic nerve fibers (Yoshida et al., 2009), noradrenaline in the LC and the NTS (Petersson et al., 1998b; Diaz-Cabiale et al., 2000a), acetylcholine in the vagal motor nucleus (Rogers and Hermann, 1985) and of endogenous opioids within in the PAG (Lund et al., 2002) and the spinal cord (Uvnäs-Moberg and Petersson, 2005). As will be discussed in detail below, the effect of oxytocin to inhibit the activity of noradrenergic neurons in the CNS is of great importance for the anti-stress effects induced by oxytocin.

### EFFECTS OF OXYTOCIN

Administration of oxytocin gives rise to many different effects. Different kinds of social interactions are promoted including mother–infant interaction and bonding between mother and infant (Keverne and Kendrick, 1994). Pair bonds may also be induced in certain types of mammals and endogenous release of dopamine is part of this process (Carter, 1998; Insel, 2003), whereas in general positive and friendly social interactions are promoted and aggression is reduced (Uvnäs-Moberg, 1998). In addition oxytocin exerts anxiolytic effects and feelings of wellbeing and reward may be induced (Uvnäs-Moberg et al., 1994). Oxytocin increases calm and the threshold to pain may be increased and the levels of inflammation may be decreased (Uvnäs-Moberg et al., 1992). Oxytocin may also induce powerful anti-stress effects by reducing the activity of the HPA-axis and of some aspects of the sympathetic nervous system, for example the activity of the cardiovascular system may be decreased. The function of the parasympathetic nervous system, and thereby the function of the gastrointestinal tract, is increased (Uvnäs-Moberg and Petersson, 2005). It should be noted that when oxytocin is released by threatening situations or an unfamiliar environment it may induce powerful protective and aggressive effects, which are also linked to an increased activity in the HPA axis and of the sympathetic nervous system (Bosch et al., 2005).

When oxytocin is administered repeatedly [subcutaneously (SC) or intracerebroventricularly (ICV)] *long-term effects* are induced. For example blood pressure and cortisol levels are decreased and pain threshold as well as the release of gastrointestinal hormones such as insulin is increased for several weeks after the last administration of oxytocin (Petersson et al., 1996a,b, 1999a,b, 2005b). These sustained effects are due to the fact that oxytocin influences the production of neurotransmitters or the function of their receptors in a long-term way. Repeated administration of oxytocin, e.g., increases the synthesis of opioids in the PAG, which is linked to the sustained elevation of pain threshold caused by oxytocin. The long term anti-stress effects caused by repeated exposure to oxytocin are linked to a changed function of mineralocorticoid receptors (MR) and glucocorticoid receptors (GR) in the hippocampus, to decreased production of corticotrophin releasing factor (CRF) in the PVN, but above all to a decreased function of central noradrenergic transmission by an increased function of inhibitory alpha 2-adrenoreceptors. Such receptors, located presynaptically on noradrenergic neurons emanating from the LC and NTS, exert an inhibitory function on the release of noradrenaline, which leads to decreased stress levels and reactivity to stress (Petersson et al., 1998b, 2005a; Diaz-Cabiale et al., 2000b; Neumann et al., 2000; Lund et al., 2002; Petersson and Uvnäs-Moberg, 2003). Other effects of repeated oxytocin administration are increased rates of learning and wound healing (Petersson et al., 1998a; Uvnäs-Moberg et al., 2000). If oxytocin is given repeatedly to rats in the postnatal period, an effect pattern similar to the one seen in adult rats is induced, the difference being that the effects might become even life-long. A decrease in blood pressure, in levels of corticosterone and an increase in nociceptive thresholds may be seen. In addition the function of central alpha 2-adrenoreceptors is increased (Sohlstrom et al., 2000; Holst et al., 2002; Diaz-Cabiale et al., 2004).

In humans, intranasal administration of oxytocin has been shown to stimulate certain aspects of social interaction, e.g., by increasing eye gazing (Domes et al., 2013) as well as the ability to interpret sensory cues such as facial expression (Domes et al., 2007) and tone of voice (Hollander et al., 2007). Also the reactivity of the amygdala may decrease, thereby reducing fear and facilitating friendly social interactions (Domes et al., 2007). It also causes anxiolytic and anti-stress effects (Heinrichs et al., 2003) and increases trust (Kosfeld et al., 2005). Oxytocin has also been shown to cause wellbeing and to decrease the experience of pain (Ohlsson et al., 2005). In other clinical trials oxytocin has, e.g., been shown to have antidepressant effects (Scantamburlo et al., 2014) and to decrease symptoms of schizophrenia (Pedersen et al., 2011), autism and Asperger syndrome (Aoki et al., 2014; Domes et al., 2014). It has also been shown to facilitate withdrawal from alcohol (Pedersen et al., 2013).

It is of importance to mention that the effect patterns induced by repeated administration of oxytocin by nasal, by ICV, SC, intravenous (IV) injections or by stimulation of endogenous oxytocin release via stimulation of sensory nerves are not always the same. For example, repeated administration of oxytocin spray to mice has been shown to result in down regulation of oxytocin receptors and to impairments in behavioral development in mice (Bales et al., 2014; Huang et al., 2014). Most likely oxytocin released in response to repeated stimulation of sensory nerves results in a more physiological and sustainable effect pattern than does any form of repeated pharmacological administration of oxytocin.

## RELEASE OF OXYTOCIN

The release of oxytocin can be stimulated by hormones such as estrogen. In addition oxytocin can be released in response to various types of sensory stimulation. Oxytocin can be released in response to stressful as well as positive and pleasant mental stimuli and in response to noxious (painful) and non-noxious (pleasant) somatosensory stimulation (Stock and Uvnäs-Moberg, 1988; Uvnäs-Moberg, 1998; Neumann, 2002; Uvnäs-Moberg and Petersson, 2005). When oxytocin is released in response to pain and stressful stimuli it may play a role in certain types of stress, and thereby it may also act to dampen stress reactions (Neumann, 2002). The non-noxious type of somatosensory stimulation is of particular importance for the hypothesis presented in this article, i.e., that oxytocin released in response to non-noxious sensory stimulation may be critically involved in many types of self-soothing behaviors, as it is linked to the effects of oxytocin associated with wellbeing and in particular reduction of stress levels, as will be described in detail below.

**FIGURE 2:**
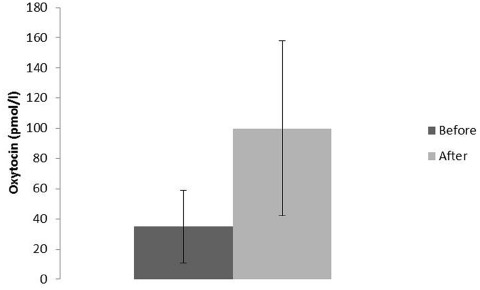
**Oxytocin plasma levels in male rats (*n* = 8) before and after 10 min of stroking (means ± SD).** Data from Stock and Uvnäs-Moberg (1988).

**FIGURE 3:**
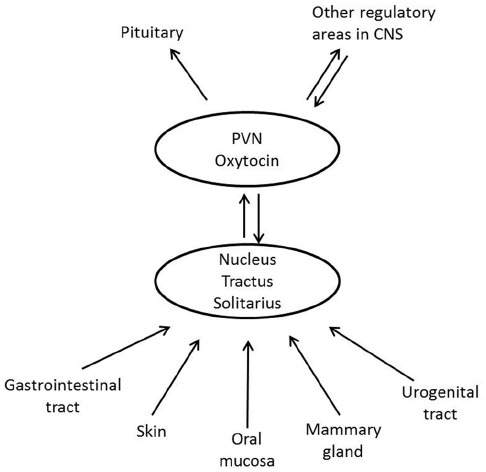
**Schematic illustration showing how afferent nerves from different parts of the body stimulate oxytocin release**.

The most well-known situations, which are related to oxytocin release, are labor and breastfeeding, when oxytocin stimulates uterine contractions and milk ejection respectively. In these situations oxytocin is released following activation of sensory nerves originating from the urogenital tract (pelvic/hypogastric nerves) and from the nipple (the mammary nerves). Oxytocin can also be released from the skin via activation of cutaneous sensory nerves in response to touch, light pressure, massage-like stroking, warm temperature and by low intensity electrical stimulation of sensory nerves in rats (Stock and Uvnäs-Moberg, 1988; Uvnäs-Moberg et al., 1993a; Lund et al., 2002) (Figure 2). Oxytocin can, however, also be released following activation of other sensory nerves originating from, e.g., the oral mucosa (Lupoli et al., 2001), and the gastrointestinal tract (vagal nerves; Stock and Uvnäs-Moberg, 1988) Irrespective of the origin of the sensory nerves involved in oxytocin release, the NTS is an important relay station for the afferent nervous impulses (Figure 3) (Burbach et al., 2006).

### NON-NOXIOUS SENSORY STIMULATION AND OXYTOCIN RELEASE

Low intensity (non-noxious) stimulation of somatosensory nerves in conscious or unconscious rats, results in increased social behavior, increased pain threshold, profound anti-stress effects, such as a decrease in blood pressure and in cortisol levels. In addition the function of the gastrointestinal tract is increased (Araki et al., 1984; Lund et al., 1999, 2002; Holst et al., 2005; Uvnäs-Moberg and Petersson, 2010). The exact nature of sensory nerves, which mediate the effects of non-noxious stimulation, is not known. Myelinated somatosensory fibers mediating the sense of touch may be involved, but a more likely candidate to this effect is the unmyelinated CT fiber afferents. In addition a subtype of vagal afferents originating in the skin on the chest may be involved (Uvnäs-Moberg and Petersson, 2010; McGlone et al., 2014). As mentioned above, non-noxious sensory stimulation results in a release of oxytocin from the SON and from the PVN. Oxytocin is not only released into the circulation, but also into the brain. In fact many of the physiological effects induced by non-noxious sensory stimulation are partly mediated by oxytocin. The increase of pain threshold induced by certain types of non-noxious sensory stimulation, e.g., warmth, is completely blocked by previous administration of oxytocin antagonists (Uvnäs-Moberg et al., 1993a; Agren et al., 1995).

A subpopulation of the oxytocinergic neurons emanating from the PVN project to the NTS and other areas in the brainstem including the LC (Buijs, 1983; Buijs et al., 1983; Sofroniew, 1983; Sato et al., 1997; Stoop, 2012). These fibers are, as described above, involved in the control of autonomic nervous tone. Most often oxytocin inhibits sympathetic nervous activity whereas it stimulates parasympathetic nervous activity (Uvnäs-Moberg, 1997). Also many physiological effects induced in the brainstem area (brainstem reflexes) in response to sensory stimulation, are modulated by oxytocinergic fibers, which originate in the PVN and project to the NTS. The oxytocin released in the NTS exerts a further important effect. As mentioned above, all types of sensory stimulation, involved in oxytocin release, relay in the NTS. Oxytocin released into the NTS from nerves originating in the PVN facilitates the transmission of sensory neurons in the NTS, which are associated with oxytocin release. In this way a feed-forward stimulation of the oxytocin release, induced by sensory stimulation, is provided (Burbach et al., 2006). Finally oxytocin, in particular after repeated administration, stimulates presynaptic alpha 2-adrenoreceptors on noradrenergic neurons originating in the NTS and LC. The firing of the neurons originating in the LC is decreased by repeated oxytocin administration. In addition administration of oxytocin increases the amount of alpha 2-adrenoreceptors in some other areas of the brain, such as the hypothalamus, the amygdala and the NTS (Petersson et al., 1998b, 2005a; Diaz-Cabiale et al., 2000b). As the alpha 2-adrenoreceptors inhibit the function of the noradrenergic neurons, which are linked to stress reactions, the oxytocin mediated increase in alpha 2-adrenoreceptor function will result in decreased stress levels and in decreased reactivity to stress. In addition gastrointestinal and metabolic functions are optimized and thereby processes related to growth (Uvnäs-Moberg and Petersson, 2005).

As non-noxious sensory stimulation increases the release of oxytocin from the oxytocinergic neurons, which project from the PVN to the LC and NTS and since oxytocin released into these areas will increase the number of alpha 2-adrenoreceptors on the noradrenergic neurons originating in the LC and NTS, it follows that non-noxious sensory stimulation will result in decreased stress levels and decreased reactivity to stress and also to stimulation of processes related to restoration and growth (Uvnäs-Moberg and Petersson, 2005). In addition the oxytocin released into the brainstem by non-noxious sensory stimulation will potentiate actions of local brainstem reflexes and also facilitate the function of sensory neurons mediating oxytocin release in the NTS (for references see above).

Oxytocin has the capacity to stimulate its own release in several ways. Oxytocin may, e.g., by activation of oxytocin receptors on the dendrites of the oxytocin producing nerves in the SON and PVN of the hypothalamus, stimulate its own release. As oxytocin is released from the dendrites of the oxytocin producing cells, a local feed forward system is activated (Ludwig and Leng, 2006). There is accumulating evidence that also circulating oxytocin can stimulate oxytocin release from the SON and PVN by activation of oxytocin receptors located on peripheral sensory nerves, such as the pelvic and the hypogastric nerves (Jonas et al., 2009). In addition oxytocin, released from the oxytocinergic neurons projecting to the NTS, may via activation of oxytocin receptors located on sensory neurons which project to the NTS, facilitate the function of these neurons thereby increasing oxytocin release (Burbach et al., 2006).

### PATHWAYS AND MECHANISMS INVOLVED IN STRESS REACTIONS AND OXYTOCIN LINKED INHIBITION OF STRESS REACTIONS

#### Mechanisms involved in stress reactions

It is well established that the HPA-axis regulates the secretion of cortisol from the adrenal glands. First CRF, produced in and released from neurons within the PVN of the hypothalamus, stimulates the secretion of ACTH (adrenocorticotropic hormone) from the anterior pituitary into the circulation. Circulating ACTH in turn stimulates cortisol secretion from the adrenal glands. Cortisol levels then exert a feedback inhibitory effect on both CRF and ACTH release (Lightman, 2008). What is less well known is that the activity of the HPA-axis is strongly influenced by noradrenaline released from noradrenergic neurons emanating from the LC and the NTS, which project to the CRF producing neurons in the PVN. The more noradrenaline that is released from these neurons, the more CRF is released within the PVN and consequently the activity of the HPA-axis is increased (Caldji et al., 2000). The activity of the noradrenergic neurons in the LC is influenced by the amygdala-hippocampal systems and the activity of the noradrenergic neurons emanating from the NTS by afferent nerves mediating noxious stimuli (Araki et al., 1984; Tsuchiya et al., 1991; Van Bockstaele et al., 1996). When the amygdala-hippocampal system is activated in response to a stressor, neurons, which project from the amygdala to the LC, are activated. (Van Bockstaele et al., 1996). Consequently the noradrenergic neurons in the LC are activated and noradrenaline is released, e.g., from the noradrenergic neurons projecting to the CRF producing neurons in the PVN. The CRF secretion is stimulated by the increased levels of noradrenaline and as a result of the increased secretion of CRF the activity in the HPA-axis is promoted. Stimulation of sensory nerves in response to dangerous or noxious sensory stimuli represents an alternative way by which the CRF secretion in the PVN and thereby the HPA-axis can be activated. In this situation noradrenergic neurons emanating in the NTS are activated by the noxious sensory stimulation and then noradrenaline released from these neurons, in turn stimulates the release of CRF (Araki et al., 1984; Tsuchiya et al., 1991).

#### Mechanisms by which oxytocin inhibits stress reactions

Oxytocin may antagonize the activity of the stress axis in multiple ways. It is well established that oxytocin released from nerves within the hypothalamus and in the anterior pituitary inhibits CRF and ACTH secretion respectively and that circulating oxytocin may inhibit cortisol secretion directly from the adrenals (Stachowiak et al., 1995; Petersson et al., 1999b; Neumann, 2002).

**FIGURE 4:**
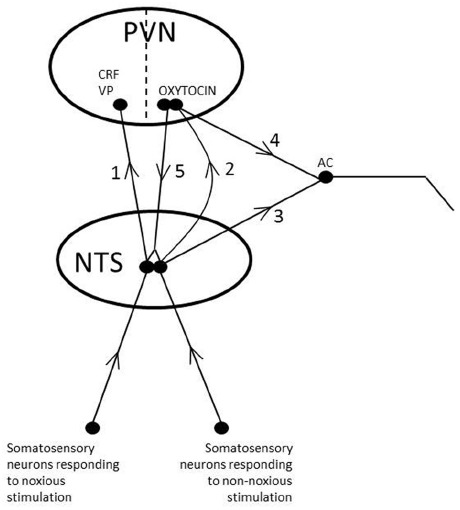
**Schematic illustration of different effects of oxytocin released from parvocellular neurons in the brainstem.** 1. Noxious stimulation of afferent sensory nerves results in release of CRF and VP from the PVN. 2. Non-noxious stimulation of afferent sensory nerves results in release of oxytocin from the PVN. 3. Noxious and non-noxious stimulation of afferent sensory nerves result in activation of sympathetic and parasympathetic neurons in the brain stem and spinal cord (autonomic centra, AC). 4. Oxytocin released from oxytocinergic neurons originating from the PVN and projecting to areas in the brainstem and spinal cord involved in the control of autonomic nervous tone (e.g., the DMX) influences the function of autonomic nervous tone. In addition it reinforces and facilitates effects caused by shorter brain stem projections. In this way hypothalamic reflexes controls and modulates the activity of the shorter brainstem reflexes. 5. Oxytocin released from oxytocinergic neurons originating from the PVN and projecting to the NTS facilitates the function of afferent neurons involved in the release of oxytocin, thereby facilitating oxytocin release. It also activates alpha 2-adrenoceptors on noradrenergic fibers innervating CRF neurons in the PVN, thereby decreasing stress reactivity. In this way non-noxious somatosensory stimulation promotes oxytocin release and oxytocin mediated effects, but counteracts CRF and VP linked effects.

Oxytocin may be released to antagonize stress reactions in three principally different ways:

1. Oxytocin is released in response to pleasant mental experiences. Such a release of oxytocin may, e.g., be induced by seeing, hearing, smelling, or thinking of well known and loved persons, but also by other pleasant situations (Uvnäs-Moberg, 1998; Uvnäs-Moberg et al., 2005).
  As oxytocin is released from neurons emanating in the PVN stress reactivity will be dampened in multiple ways. The activity in the HPA-axis will be reduced by oxytocin according to the pattern described above. In addition, as oxytocin is released from neurons within the amygdala, the reactivity to fear and stress is dampened and consequently the activity of neurons that project from the amygdala to the LC will be decreased (see above). As a consequence of a less intense stimulation of the function in the LC, the release of noradrenaline from the noradrenergic neurons emanating in the LC declines. As less noradrenaline is released from the nervous projection to the PVN, less CRF is secreted and consequently the activity in the HPA-axis is diminished. Also the activity in other areas involved in stress regulation and which are receiving noradrenergic projections from the LC will be decreased. The activity in the LC and NTS will of course also be decreased by oxytocinergic neurons projecting directly to these areas.
2. Oxytocin is also released in response to activation of somatosensory nerves, which mediate non-painful and pleasant (non-noxious) information, e.g., induced by touch, stroking, warmth, and light pressure of the skin (Uvnäs-Moberg and Petersson, 2010).
  Oxytocin may in response to non-noxious stimulation be released into the hypothalamus to reduce the activity in the HPA-axis and into the amygdala to decrease the reaction to stress and fear and thereby the activity of the noradrenergic neurons in the LC, which control the activity of the HPA axis. In addition, oxytocin released from neurons projecting from the PVN to the LC and NTS in the brainstem, may decrease stress reactions by reducing the activity in the stress related noradrenergic neurons emanating from these nuclei. The effect of oxytocin released from the neurons that project from the PVN to the NTS involves activation of alpha 2-adrenoreceptors, which inhibit the function of the noradrenergic neurons in the LC and NTS. In this way the secretion of CRF in the hypothalamus and the activity of the HPA- axis are further decreased, as described in detail above. Taken together oxytocin release induced by non-noxious somatosensory stimulation inhibits stress by direct actions in the amygdala, the hypothalamus, the LC and the NTS. It, however, also acts more indirectly by decreasing the function in the noradrenergic pathways emanating in the LC and in the NTS, which project to the PVN and exert an inhibitory function on the HPA-axis (Figure 4).
3. In addition, oxytocin may also be released by mental and sensory stimuli that are perceived as stressful. In this case oxytocin is activated in parallel with the stress system and the role of oxytocin in these situations may be to dampen stress responses and facilitate coping behaviors (Neumann, 2002).

#### Oxytocin release in response to non-noxious sensory stimulation is linked to potent anti-stress effects

The oxytocin release caused by non-noxious somatosensory stimulation gives rise to particularly pronounced anti-stress effects because:

1. Oxytocin released into the NTS and DMX reinforces the effect of local brainstem reflexes, e.g., involving regulation of sympathetic and parasympathetic nervous tone, which leads to decreased blood pressure and pulse rate and increased gastrointestinal function (Sato et al., 1997).
2. Oxytocin released in the NTS and LC stimulates the production of alpha 2- adrenoreceptors on noradrenergic neurons projecting to the CRF neurons in the hypothalamus and to the sympathetic nervous centers (Petersson et al., 1998b, 2005a). It is also possible that the sensory neurons that synapse in the NTS modulate the activity of the alpha 2-adrenoreceptors or other oxytocin binding receptors on noradrenergic neurons thus making these receptors more sensitive to the effects of oxytocin.
3. Oxytocin released within the NTS facilitates the transmission of incoming sensory nerves involved in the release of oxytocin, thereby inducing a feed forward effect on oxytocin release (Burbach et al., 2006).

## OXYTOCIN RELEASE AND EFFECTS DURING LABOR AND BREASTFEEDING

As mentioned above it is well known that oxytocin is released during labor and breastfeeding to induce uterine contractions and milk ejection. The intense stimulation of the pelvic/hypogastric nerves and the mammary nerves respectively induced in theses situation causes a release pattern characterized by short pulses (Fuchs et al., 1991; Nissen et al., 1996; Jonas et al., 2009). Still the concomitant effect pattern differs between the two situations. During labor the pulsatile oxytocin release is associated with high stress levels and cortisol levels are elevated and blood pressure is high in order to make the hard work of labor and uterine contractions possible. During breastfeeding, on the other hand, cortisol and blood pressure fall in response to each suckling episode (Nissen et al., 1996; Uvnäs-Moberg, 1996; Handlin et al., 2009). Oxytocin is also released into the brain during labor and breastfeeding. Oxytocin thereby induces pain relief during labor and also helps to adapt the mothers to the role of motherhood by increasing social skills and by decreasing anxiety (Nissen et al., 1998; Jonas et al., 2008a).

### LONG-TERM HEALTH PROMOTING EFFECTS IN BREASTFEEDING MOTHERS

Research has shown that mothers, who have breastfed for several weeks have lower basal, systolic and diastolic, blood pressure (Jonas et al., 2008b) and also lower stress reactivity (as long as they feel safe and there is no threat to the baby) than women of the same age, who are not pregnant or breastfeeding. They are also calmer and more socially inclined (Nissen et al., 1998; Jonas et al., 2008a). These long-term adaptive changes are most likely induced by a changed function in other signaling systems as a consequence of the repeated exposure to endogenous oxytocin that occurs during breastfeeding. As mentioned above repeated administration of oxytocin changes the function of several types of signaling systems in the brain in a long-term way. The findings of a reduced risk for certain kinds of cardiovascular disease and also of diabetes type 2 many years after the end of breastfeeding in mothers who have breastfed several children for long periods of time supports the existence of a link between repeated exposure to endogenous oxytocin and long-term anti-stress and health promoting effects (Lee et al., 2005; Stuebe et al., 2005). An increase in the function of alpha 2-adrenoreceptors may play a pivotal role in these health promoting effects by decreasing stress levels and stress sensitivity.

## OXYTOCIN RELEASE AND EFFECTS BY SKIN-TO-SKIN CONTACT

Oxytocin cannot only be released by the suckling stimulus but also by stimulation of cutaneous nerves, As mentioned previously oxytocin has in experiments performed on rats been demonstrated to be released in response to several types of non-noxious sensory stimulation, such as touch, massage, stroking, warm temperature, and low intensity electrical stimulation. Stimulation of cutaneous sensory nerves is an important and common aspect of many types relationships, e.g., between mother (father) and infants, between human adults involved in positive relationships and even between humans and dogs. Therefore oxytocin release and oxytocin mediated effects caused by pleasant sensory stimulation should play an important role in all these types of relationships. The release of oxytocin and the pattern of oxytocin mediated effects induced during skin-to-skin contact between mother and infant after birth will be described in some detail, as this type of interaction could be regarded as archetypal representation of interactions between humans or between humans and animals, involving close physical contact.

### OXYTOCIN RELEASE DURING SKIN-TO-SKIN CONTACT BETWEEN MOTHER AND INFANT IMMEDIATELY AFTER BIRTH

Oxytocin is for example released into the maternal circulation in response to skin-to-skin contact between mother and infant immediately after birth. The oxytocin pulses induced by skin-to-skin contact are more long lasting than those observed during labor and breastfeeding. The maternal release of oxytocin is induced by activation of sensory nerves in the skin, which are activated by touch, warmth, and stroking in connection with skin-to-skin contact with the baby and also by massage-like hand movements performed by the baby. (Nissen et al., 1995; Matthiesen et al., 2001). The skin-to-skin contact between mother and infant after birth is linked to an increase in maternal, vocal and tactile interaction with the child. In addition the mother looks and smiles more at the baby (Velandia et al., 2012). At the same time anti-stress effects are induced as the mother becomes calmer and cortisol levels drop (Handlin et al., 2009). Oxytocin released into the brain is likely to lie behind these behavioral and physiological effects induced by skin-to-skin contact (Uvnäs-Moberg and Prime, 2013).

The newborn infant produces its own oxytocin. If oxytocin levels are measured in both mothers and infants directly after vaginal birth, the infant’s oxytocin levels are in fact higher than those in the mother (Marchini et al., 1988). Skin-to-skin contact after birth is in newborns, like in the mother, associated with increased social interaction. The newborns perform a spontaneous breast seeking behavior (breast crawling) and they vocalize more than infants not being allowed skin-to-skin contact. In addition they become calmer and stop crying (Widstrom et al., 1987; Christensson et al., 1995). Powerful anti-stress effects are induced; cortisol levels fall, pulse rate becomes regularized and skin temperature increases as a sign of decreased sympathetic nervous tone (Bystrova et al., 2003; Bergman et al., 2004; Uvnäs-Moberg and Prime, 2013).

Birth is a stressful event and high stress levels are of importance for the baby during birth and also for some postnatal, physiological adaptations to occur. It is of equal importance to dampen the high stress levels as soon as possible after birth. This conversion from a state of stress to a state of calm is induced in a natural way by the skin-to-skin contact with the mother immediately after birth and in this way skin-to-skin contact after birth serves to reverse the stress of being born (Bystrova et al., 2003). Also the positive effects observed on bonding between mother/father and infant and on the maturation and growth of premature infants being cared for by kangaroo care are in part linked to oxytocin being released by the close contact (Uvnäs-Moberg and Prime, 2013).

The high levels of oxytocin, which are induced during labor, are of importance for oxytocin release and oxytocin mediated effects caused by skin-to-skin contact and suckling after birth. This is demonstrated by the finding that skin-to-skin contact and suckling fail to induce any oxytocin release in mothers who have been subjected to an elective Cesarean Section, because these mothers have not been exposed to oxytocin release during labor (Velandia, 2012). These mothers do not have any oxytocin release since there was no labor. As described in detail above, oxytocin released from the PVN into the NTS increases the release of oxytocin, in response to somatosensory stimulation, by facilitating the function of incoming sensory nerves involved in oxytocin release (for references see above). When no oxytocin has been released to “open the gate” for incoming neurogenic impulses, skin-to-skin contact and suckling fail to release oxytocin. If, however, these mothers are given an infusion of exogenous oxytocin after birth (postpartum), the effect of skin-to-skin contact is restored and the mothers do release oxytocin in response to skin-to-skin contact and suckling (Velandia et al., 2012). Another consequence of elective Cesarean section is that the psychological maternal adaptations normally induced after labor, e.g., increased levels of social interaction and decreased levels of anxiety, do not develop, as no release of oxytocin occurs in the brain during birth (Nissen et al., 1998; Velandia et al., 2012). Oxytocin infusions postpartum, restore the maternal psychological adaptations (Velandia, 2012).

### LONG-TERM EFFECTS OF SKIN-TO-SKIN CONTACT

It is well known, since the work of Klaus and Kennell, that close contact between mothers and infants immediately after birth, i.e., during the early sensitive period, promote future interaction between the mother and infant (Kennell et al., 1975). Both mothers and infants having had 2 h of skin-to-skin contact after birth were shown to interact better with each other and the infants were shown to handle stress better 1 year later, than did mothers and infants that were separated after birth. The immediate oxytocin promoted or facilitated effects on social interactive behaviors and stress reactivity that was induced during skin-to-skin after birth became sustained and expressed in a more developed or mature way 1 year later (Bystrova et al., 2009).

Data from animal experiments support these findings and also extend the results from a mechanistic point of view. If newborn rats are exposed to extra sensory stimulation of the skin by intense maternal licking (or by brushing), the animals become more social and less anxious and stressed as adults (Francis et al., 1999; Holst et al., 2002). The physiological and behavioral changes induced by tactile stimulation in the newborn rats are associated with changes in the function of several transmitter systems. The increased levels of social performance and the decreased levels of anxiety are linked to an increased amount of oxytocin receptors in the amygdala (Francis et al., 2000). Reduced levels of CRF in the PVN of the hypothalamus and an increased number of alpha 2-adrenoreceptors in the NTS and the LC also contribute to the long-term anti-stress pattern induced by tactile stimulation in the newborn period. The increased function of alpha 2-adrenoreceptors is caused by oxytocin released into the NTS and LC from oxytocinergic nerve fibers emanating from the PVN, which are activated in response to tactile stimulation. In this way, as described before, stress reactions are blunted because the activity of noradrenergic fibers originating in the NTS and LC is reduced. As the levels of noradrenalin, which is of importance for the secretion of CRF in the PVN, decrease, the activity in the HPA axis will be reduced. Interestingly, the more tactile stimulation rat pups had been exposed to postnatally, the higher the amount of alpha 2-adrenoreceptors were in the adults rats (Caldji et al., 2000). Data, showing that newborn rats having received multiple doses of oxytocin, have lower cortisol levels and blood pressure, higher pain thresholds and more alpha 2-adrenoreceptors as adults, support the important role for oxytocin in these early adaptive changes (Uvnäs-Moberg, 1998; Diaz-Cabiale et al., 2000b; Sohlstrom et al., 2000; Holst et al., 2002). All the long-term effects described above, caused by tactile stimulation seems to be induced by early epigenetic programming (Cameron et al., 2008).

It is well known that early exposure to stress, during pregnancy or in early life, may lead to lifelong sensitivity to stress and stressors and also to an inhibition of social interactive behaviors (Anacker et al., 2014). This pattern is, e.g., linked to an enhanced activity in the HPA-axis and the sympathetic nervous system. CRF and also vasopressin in the hypothalamus and noradrenergic neurons emanating in the LC and the NTS are important regulators of this stress reaction/pattern. The data described in this article suggests that also a partly opposite effect pattern exists, which is induced by non-noxious somatosensory stimulation. This pattern can be learnt or imprinted early in life and is linked to high levels of friendly social interaction and low stress reactivity. This system is linked to a moderate activity in the HPA-axis and in the sympathetic nervous system and in addition to a high activity in the parasympathetic nervous system. Oxytocinergic nerves emanating from the PVN are of importance in the regulation of this “calm and connection” system (for overview, see Uvnäs-Moberg, 2003, 2013).

### LINK BETWEEN SKIN-TO-SKIN CONTACT, RELEASE OF OXYTOCIN, AND DEVELOPMENT OF SECURE ATTACHMENT

The long-term effects of skin-to-skin contact described above, i.e., increased social interaction and enhanced ability to handle stress, as observed by the PCERA (parent–child early relationship assessment) method (Bystrova et al., 2009), display some similarities with the expressions of secure attachment, as reflected in the strange situation test (Ainsworth, 1989). An interesting question is therefore, whether secure attachment is associated with a well-functioning oxytocinergic system? If so, stimulation of oxytocin release in response to skin-to-skin contact or other types of closeness, especially if induced repeatedly and early in life, could be an important mechanism, through which secure attachment is developed. The oxytocin release and the positive effects of skin-to-skin contact, originally triggered, by the cutaneous sensory stimulation in connection with skin-to-skin contact, may by time be developed into “conditioned reflexes.” After a while just seeing, hearing, or smelling the mother may trigger oxytocin release in the infant in a Pavlovian manner. Further on just the thought of or the mental image of the mother may be enough to trigger oxytocin release in the child. In conclusion the more closeness, physical as well as mental, a child receives the more the function of the oxytocin system will be stimulated. In the end those individuals, who have a well-developed oxytocin system will interact with others in a secure and trustful way and their ability to handle stressful situations will be optimized (Uvnäs-Moberg, 1996, 2006; Tops et al., 2007, 2014). The findings in some studies of higher oxytocin levels in individuals with secure attachment than in those with insecure attachment support the role of a well-functioning oxytocin system in individuals with secure attachment (Tops et al., 2007; Gordon et al., 2008). In addition administration of oxytocin spray has been demonstrated to enhance the experience of attachment security (Buchheim et al., 2009).

## CONSEQUENCES OF A LOW FUNCTION IN THE OXYTOCIN SYSTEM

From the perspective of this article it is of particular interest that some individuals with insecure attachment do not only have lower levels of oxytocin than those with secure attachment, they also have an increased risk of developing certain symptoms and diseases. Individuals having insecure attachment more often report high levels of anxiety, depression and stress than those who are securely attached (for a review, see Julius et al., 2013). In addition they have an increased risk of pain and inflammation (Davies et al., 2009). For example women with insecure attachment more often have pain during labor and during intercourse (dyspareunia; Granot et al., 2011; Costa-Martins et al., 2014) than those who have secure attachment. As oxytocin released within the brain from nerves emanating from the PVN is involved not only in the regulation of social interaction and anxiety, but also of pain and inflammation, a low function in the oxytocinergic system could underlie or at least contribute to the expression of these symptoms in individuals with insecure attachment (for references, see above).

In addition a disturbed function of the oxytocin system has been demonstrated in certain medical conditions, which may in fact to a certain extent overlap with insecure attachment. Low levels of oxytocin have, e.g., been demonstrated in individuals with borderline disease, certain types of depression and schizophrenia (for a review, see Kim et al., 2013). Also some pain syndromes such as fibromyalgia and recurrent abdominal pain in children are associated with low levels of oxytocin (Alfven et al., 1994; Anderberg and Uvnäs-Moberg, 2000). In addition, previous experience of traumatic events is associated with an increased incidence of low oxytocin levels or stress related reduction of oxytocin levels (Pierrehumbert et al., 2010).

It is, however, important to mention that low and high levels of oxytocin could not always be categorized as bad or good. As peripheral oxytocin levels and also the effects of oxytocin are influenced by many different factors, other relationships also exist (Bartz et al., 2011). For example, as will be described more in detail later in this chapter, oxytocin levels display two peaks during encounters with other individuals, one when meeting and approaching the other individual and one when being in close contact and receiving sensory stimulation by the other individual. The first peak is linked to arousal and an increased activity in the stress axis and the second peak is linked to decreased stress levels (Rehn et al., 2014). From this perspective a high oxytocin level could represent either a “frustrated approach or reward seeking” aspect of oxytocin or the “satisfied calm” aspect from having received the rewarding sensory stimulation.

## OXYTOCIN RELEASE IN POSITIVE HUMAN RELATIONSHIPS

As mentioned above oxytocin levels may rise as a consequence of closeness between mothers and infants, and when they are bonded or attached to each other, oxytocin levels rise also when they just see, hear or even think of each other (Swain et al., 2007; Kim et al., 2014).

A similar reaction takes place in adults as warm partner contact has been demonstrated to be linked to oxytocin release and anti-stress effects. In fact oxytocin may be released, when individuals of both sexes and all ages touch each other, given that the relationship is perceived as positive. Oxytocin may even be released by seeing, hearing or by merely thinking of the other beloved person (Carter and Keverne, 2002; Grewen et al., 2005; Light et al., 2005; Holt-Lunstad et al., 2008). In stable long-term relationships oxytocin levels may display a chronic rise and some studies show that basal oxytocin levels are higher in individuals who live together. The high levels of oxytocin are most likely a consequence of cohabitation, but it can of course not be excluded that individuals who have high oxytocin levels more often choose to cohabitate than those with low oxytocin levels (Humble et al., 2013).

Many studies demonstrate that the health profile of people, who live in good relationships, is better than for those who live alone. They, e.g., have lower blood pressure and a decreased risk for cardiovascular disease. They have less infections and the risk for some types of cancer is reduced. People who live in good relationships may even look younger and live longer than those, who live alone. It is however of importance to note that the relationship should be warm and of a good quality for these positive health consequences to develop. Relationships characterized by fear and distrust, may give rise to an increased risk of cardiovascular disease in particular in women (Seeman, 1996; Uchino and Garvey, 1997; Christenfeld and Gerin, 2000; Blom et al., 2003; Danoff-Burg and Revenson, 2005).

### OXYTOCIN AND SEX

Sexual relationships are linked to oxytocin release. Data from both animals and humans demonstrate that large amounts of oxytocin are released in response to sexual activity both in females and males. In humans the peak of oxytocin seems to coincide with orgasm (Carmichael et al., 1987). Oxytocin has been demonstrated to promote sexual functioning and has been shown to increase the drive for sex, to facilitate ejaculation and transport of eggs, and the experience of orgasm in both men and women. In addition, oxytocin has been demonstrated to be linked to bonding between individuals induced by sex but also to the reduction of anxiety and increased wellbeing and calming induced by intercourse (Carmichael et al., 1987; Anderson, 2006). Long-term studies suggest that individuals with a good sex life are healthier and live longer, than those without it. It is likely, that the oxytocin release during sex contributes to the health promoting effects, but the effects could also be indirectly mediated by the strengthening of the relationship and of the bonding that is often the consequence of a good sex life (Brody, 2010).

## OXYTOCIN RELEASE IN RESPONSE TO INTERACTION BETWEEN HUMANS AND ANIMALS

Oxytocin levels peak significantly in both dog owners and dogs when they interact and in particular when the owner strokes and caresses her dog (Odendaal and Meintjes, 2003; Miller et al., 2009; Handlin et al., 2011). Oxytocin is however also released when the dogs see and want to approach the owner. These two separate phases of oxytocin release are described in the following experiments. Dogs were exposed to a short separation from a familiar person and then reunited with this person after 30 min. When the familiar person returned, the mere sight of her induced a peak shaped oxytocin release as well as an increase in cortisol levels in the dogs. After the reunion the familiar person either ignored the approaching dogs or had verbal contact without touching them, or the familiar person both talked to and had physical contact with the dogs. Only in the third scenario, where the dogs received physical contact from the familiar person, did oxytocin levels continue to be elevated. In addition, cortisol levels decreased in these dogs demonstrating that oxytocin release induced by tactile stimulation is associated with anti-stress effects. In the two other scenarios where the familiar person did not touch the dogs, because they ignored them or just had verbal contact with them, no further oxytocin was released and the dogs cortisol levels remained high (Rehn et al., 2014).

In another experimental setting, children with severe attachment problems were exposed to a stressful task. Cortisol levels were measured in saliva in order to monitor the stress reaction. When these children performed the stress test they were allowed support either by a trained and emphatic human, a friendly therapy-dog dog or a stuffed toy dog. The results showed that salivary cortisol, was significantly lower in the children, who had a friendly therapy-dog as a companion during the stress test, compared to the children who had a friendly human or a toy dog. In addition, the lowering of cortisol levels seen in the children who had a dog present during the stress test correlated with the amount of physical contact between the child and dog (Beetz et al., 2012). The finding that humans failed to calm the child and lower their cortisol levels may be related to the fact that the children with severe attachment problems were too afraid of humans in order to receive any support from them. In contrast, interaction with the living therapy-dog was accompanied by decreased cortisol levels. As stroking releases oxytocin and since oxytocin decreases the release of cortisol, the decrease in cortisol levels observed in the boys that were physically interacting with the dogs are likely to be secondary to a release of oxytocin triggered by touch. The stuffed dog did not give rise to the same stress buffering effects as the real dog. This may be due to the fact that just seeing and approaching the friendly therapy-dog triggers a peak of oxytocin in the child just as it did in dogs when they were reunited with their familiar person, as described above. When meeting a stuffed dog this “startup” peak of oxytocin was absent and stroking of the toy dog did not as efficiently trigger oxytocin release and as a consequence no decrease in cortisol levels was induced.

Taken together these two experiments regarding interaction between humans and dogs demonstrated that interaction between the two individuals comprises of two separate phases of oxytocin release. The first oxytocin peak is induced when seeing and hearing “the other individual” (dog or the human) and is linked to active approach. If the approach phase is followed by physical interaction a second phase of oxytocin release is activated. This second phase of oxytocin release is associated with a reduction of stress levels, e.g., lowered cortisol levels. Still the two phases, the approach and the interaction/closeness phase, are not completely independent of each other. Physical interaction with a toy dog did not result in the same stress reduction (as a consequence of oxytocin release), as did the real dog. This is probably due to the fact that the stuffed dog was not as attractive as the real dog and in the absence of a joyful approach phase no oxytocin release was induced. In contrast the real dog triggered an initial pulse of oxytocin. This situation is analogous to the previous observations of presence or absence of oxytocin release in response to skin-to-skin contact depending on the type of birth, as described above. During normal vaginal labor oxytocin is released and oxytocin is released following skin-to-skin contact after birth. In the absence of oxytocin release during birth, as, e.g., after an elective Cesarean section, no oxytocin will be released by skin-to-skin contact or suckling in the postpartum period (Velandia et al., 2012). However, this effect was restored in mothers who had received infusions of exogenous oxytocin postpartum.

This two phase model of oxytocin release during interactions as described above is most likely not only related to interactions between dogs and humans but is of general importance and is most likely also present during interaction between humans in different situations where, e.g., skin-to-skin contact or touch in any type of situation is promoted by previous oxytocin release. As described above, previously induced oxytocin release into the NTS opens up for further oxytocin release in response to tactile stimulation and closeness. It should be noted that this two phase release of oxytocin occurs as long as the individual is experiencing the situation in a positive way. If the situation is experienced as stressful or threatening the release of oxytocin will be absent.

### POSITIVE RELATIONSHIPS BETWEEN HUMANS AND DOGS ARE ASSOCIATED WITH HEALTH

Stroking of a dog has been shown to significantly reduce blood pressure and cortisol levels (Odendaal and Meintjes, 2003; Miller et al., 2009). In addition, pet ownership is associated with lower blood pressure, serum triglycerides, and cholesterol levels (Allen et al., 2002). Patients suffering from a heart attack had significantly higher 1-year survival rates if they had a pet, compared to those without pets, and dog owners are 8.6 times more likely to be alive after 1 year (Friedmann et al., 1980; Friedmann and Thomas, 1995). Pets also have a positive impact on the ability to cope with chronic conditions and on the course and treatment of illness such as heart disease, dementia, and cancer (Johnson et al., 2003; Friedmann and Tsai, 2006). Recovery of hospitalized children has been facilitated by interaction with companion animals (Walsh, 2009) and the animals also ease suffering and anxiety at the end of life for those in palliative and hospice care (Geisler, 2004). In addition, children having a dog present in their classroom display increased social competence and concentration (Hergovich et al., 2002; Kotrschal and Ortbauer, 2003).

Studies investigating the effects of interaction between dog owners and their dogs in a more long-term perspective indicate that there is a mutual relationship between owners and their dogs, where positive (or absence of negative) aspects of the relationship are linked to higher oxytocin levels in both species. In addition, frequent sensory interaction between dog-owners and their dogs was associated with higher oxytocin levels in both species. It is likely that the increased interaction generates the higher oxytocin levels, but it can of course not be excluded that “high oxytocin individuals” interact more (Handlin et al., 2012).

## OXYTOCIN RELEASE AND EFFECTS BY MASSAGE

The release of oxytocin normally occurring in response to closeness in good relationships can to a certain extent be mimicked by massage and stroking of the skin. Indeed, treatment with massage is linked to oxytocin release. If repeated blood samples are collected in the beginning of a massage session, pulses of oxytocin can be observed both in the individual receiving massage and in the masseur (Uvnäs-Moberg, 2004). The massage treatment is accompanied by several positive effects. During a massage session levels of anxiety are decreased, the perception of wellbeing is increased and that of pain decreased. Moreover, both blood pressure and cortisol levels are lowered. Repeated massage treatments are associated with long-term expression of all these effects (Field, 2002, 2014). Massage also increases the ability for friendly interaction, and may even be used to resolve marital conflicts (Ditzen et al., 2007). Infant massage has been shown to decrease maternal depression, to ameliorate bonding between mothers and infants and also to relieve stress reactions and colic in the infants (O’Higgins et al., 2008; Field et al., 2009). Oxytocin, released into the brain in response to the massage, should be an important mediator of the above, mentioned effects.

## OXYTOCIN RELEASE IN RESPONSE TO FOOD INTAKE

Food intake is also associated with oxytocin release and several mechanisms are involved in the oxytocin release induced by ingested food. When food touches the oral mucosa oxytocin is released following activation of touch receptors in the oral cavity and when the ingested food reaches the gastrointestinal tract, the gut hormone cholecystokinin (CCK) is released from the duodenum in particular in response to proteins and fat. Sensory fibers of the vagal nerves are then activated by CCK. The sensory vagal nerve fibers relay in the NTS wherefrom neurons project to the PVN, where oxytocin is released both into the circulation and into the brain (for references, see Uvnäs-Moberg and Prime, 2013).

Suckling is also linked to oxytocin release as the act of suckling *per se* induces oxytocin release by activation of touch receptors in the oral cavity (Lupoli et al., 2001). Oxytocinergic mechanisms may be involved in the calming, anti-stress, and growth promoting effects of suckling in breastfeeding infants, but also in response to sucking of a pacifier (Uvnäs-Moberg et al., 1987). Also the attachment between infant and mother may in a primitive sense be linked to oxytocin release caused by suckling. It is even possible that the dependency of other types of suckling related behaviors, e.g., smoking of cigarettes and even drinking of alcohol (Uvnäs-Moberg et al., 1993b), may involve an oxytocin linked component triggered by the suckling itself and not only by the pharmacological effects of nicotine and alcohol.

Food intake is followed by “postprandial sedation” or a state when people feel calm and satisfied in a broad sense and often open up for social interaction and even bonding and attachment. Some of these effects involve oxytocinergic mechanisms (Uvnäs-Moberg, 1994). As eating has apparent rewarding and also relaxing effects, it represents an important pathway to achieve wellbeing and stress relief and eating or overeating for self-soothing is very common. Unfortunately overweight and obesity are long-term consequences of overeating.

## GENERAL DISCUSSION

A common denominator for breastfeeding, skin-to-skin contact between mothers and infants, warm interactions with or without sex between adults, as well as tactile interaction with a friendly or beloved dog and even massage or other types of tactile treatments which represent different kinds of relationships with other living beings, is that they are accompanied by oxytocin release through activation of sensory nerves. Thereby a number of oxytocin mediated effects, such as increased social interactive behaviors, wellbeing, pain relief and anti-stress effects are activated. In addition attachment/bonding, which involves oxytocinergic mechanisms, may develop. These situations linked to sensory stimulation and oxytocin release are also in a long-term perspective linked to good mental and physical health. Breastfeeding is linked to a “dose-dependent” decrease in cardiovascular disease and diabetes mellitus type 2 in the mothers. Skin-to-skin contact between mothers and infants is linked to increased social interaction and calm. In addition, closeness in early life may promote the development of secure attachment. Adult individuals with secure attachment display less anxiety and depression, less pain and inflammation and a reduced risk for cardiovascular disease. A similar positive effect spectrum has been demonstrated for adult individuals, who are engaged in warm and positive relationships including having a good sex life, and they also live longer. Interestingly the same positive health pattern emerges in individuals having a pet, e.g., a dog. Dog owners seem to be protected from certain types of stress related diseases such as cardiovascular diseases and have lower blood pressure.

Taken together, positive relationships irrespective of age and sex in particular if they involve closeness serve to create wellbeing and reduce stress levels on an everyday basis. Some individuals may prefer to relate with animals rather than relating to humans and a good relationship with a dog that involves physical contact seems to be an efficient way to achieve wellbeing, stress relief, and health promoting effects. These types of everyday interactions serve as positive self-soothing behaviors. In the absence of close and well-functioning social relationships with humans or dogs, alternative pathways may be used to achieve wellbeing and stress relief. Interestingly massage or other types of tactile interventions give rise to similar effect spectrums as the positive interactions listed above and can therefore be used to increase wellbeing, reduce anxiety and stress levels and increase social interactive behaviors. Also yoga, different types of relaxation techniques as well as physical exercise is used by many people to achieve such positive effect. Some other types of “interactions” with ingested or inhaled material, e.g., food, cigarettes, and even alcohol, may also be used to compensate for the rewarding actions normally achieved in a good interpersonal relationship. The calming and in a broad sense satisfying effects obtained after food intake are available to everybody. To use ingestion of food as a way to feel good and to reduce stress levels is very common in our time, and may result in overeating, and obesity. The difficulties in breaking too generous feeding habits may in part be due to an oxytocin mediated bonding to the food. Similar mechanisms may be involved in smoking and even ingestion of alcohol.

The activity of the function of the oxytocin system differs between individuals, too much or too little oxytocin may be secreted and oxytocin receptors may be deficient. Such differences may be of genetic or epigenetic origin. Individuals, who for various reasons have a low function in their oxytocin system, may have an insecure type of attachment, have problems with relationships or feel depressed or anxious for other reasons, may feel a stronger need than others to improve their mood and to relieve feelings of stress, tension or pain. Different individuals use different routes to reach these goals. Some people indulge in too much sex, others eat too much. Both these behaviors will in the long run lead to negative effects regarding the ability to form positive relationships or to retain a normal weight.

### Conflict of Interest Statement

The authors declare that the research was conducted in the absence of any commercial or financial relationships that could be construed as a potential conflict of interest.
